# Evaluation the Efficiency of Electrical Stimulation Advanced Methods on Management of Bowel and Bladder Functions in Spinal Cord Injury Subject; A Systematic Review of Literature

**DOI:** 10.30476/BEAT.2021.89300.1227

**Published:** 2022-01

**Authors:** Abolghasem Fallahzadeh Abarghuei, Mohammad Taghi Karimi

**Affiliations:** 1 *Occupational Therapy Department, Faculty of Rehabilitation, Shiraz University of Medical Sciences, Shiraz, Iran*; 2 *Rehabilitation Sciences Research Center, Shiraz University of Medical Sciences, Shiraz, Iran*

**Keywords:** Spinal cord injury, Neurogenic bowel, Urinary bladder, Electrical stimulation.

## Abstract

**Objective:**

To evaluate the efficiency of various methods used for SCI subjects in this regard based on the available literature.

**Methods:**

A search was done in some data bases such as Google scholar, ISI web of knowledge, PubMed, and Scopus. Some keywords such as bowel, bladder control and management were used in combination with SCI. The studies’ quality was evaluated with Pedro scale.

**Results:**

From 100 articles found, 21 papers were selected based on abstracts and titles. The quality of the studies varied between 5 and 7 based on Pedro scale. There were 3 studies on abdominal muscles stimulation, 1 on stimulation of tibial nerve, 8 on stimulation of sacral nerve root, 2 on combination of stimulation and exercise, 4 on Brindley bladder control and 3 on sacralizotomy.

**Conclusion:**

The bowel and bladder management functions is not the main problem of SCI subjects anymore. Some advantages of the mentioned procedures used for SCI subjects are including improved quality of life, socialization, and decreased bladder infection.

## Introduction

Spinal cord injury (SCI) is defined as damage to spinal cord which results in loss of function, sensation and mobility depends on level of injury [1]. The injury incidence varied from a country to another country between 14 and 55 new cases per million populations each year [1-4]. These subjects miss their abilities to stand and walk and have to use various assistive devices to restore their abilities to walk and to ambulate from a place to place [5-8]. They suffer from joint contracture, inability to control bowel and bladder functions, problems with digestive system and cardiovascular impairment [9]. 

Various treatment approaches have been used for SCI to restore their abilities to stand and walk [6, 7]. However, bowel and bladder function control is another issue which should be emphasized [9-13]. Elbert study showed that bowel problems occur in 27% to 62% of the patients with SCI [14]. Moreover, the time spent to manage bowel and bladder is another important issue. Bowel and bladder function and automatic dysreflexia elimination seems to be the 1^st^ and 2^nd^ highest priorities for 39.7% and 38% of quadriplegia and paraplegia, respectively [15]. It should be emphasized that impaired bladder emptying is one of the important factor of urinary infection and stone formation, which finally lead to hydronephrosis and renal damage. Different approaches have being used to help SCI individuals to manage their bowel and bladder functions includes conventional method, stimulation of sacral nerve or abdominal muscles and surgery [11, 12, 16-19]. Intermittent catherization, indwelling catherization, manual expression and use of reflex bladder contraction are some of the conventional methods used in this regard [20]. Bowel management should be used for the subjects with SCI in order to achieve regular and predictable bowel empty at social acceptable time and place, avoid constipation and faecal incontinence and to manage bowel within reasonable time. Actually, various steps have been used to manage bowel and bladder functions includes change in dietary patterns and life style, abdominal massage, oral laxatives, digital anal stimulation, anal plugs, trans anal irrigation and electrical stimulation therapies.

Electrical stimulation includes magnetic stimulation use of sacral nerve or deep muscles which has been used to improve the social performance of bowel and bladder system [21, 22]. Various approaches have been recommended to be used for the subjects with spinal cord injury to manage their bowel and bladder functions. There are some reviews on the efficiency of diet and medicine use which supports the effects of these approaches on bowel and bladder functions management. However, the effects of other types of interventions are still controversial. Therefore, the main question posted here is that: which one of the aforementioned methods, except medicine and diet can be used successfully for this group of subjects to enhance their bowel and bladder function control in a social way. The purpose of this study was to determine the most user friendly approach of bowel and bladder control in SCI subjects based on the available literature.

## Materials and Methods

A search was done in some data bases include PubMed, ISI web of knowledge, Scopus, and Google scholar between 1960 and 2020. Some keywords such as bowel and bladder management, functional electrical stimulation (FES), magnetic stimulation, exercise therapy, manual bowel and bladder management were used in combination with spinal cord injury. The papers were selected based on their titles and abstracts. Finally the papers were selected based on the following criteria:

1. The papers were published in English

2. Focus on the aforementioned key words

*Type of Studies*

Although the emphasize of this study was to focus mostly on randomized control trial (RCTs), due to lack of these studies on this topic, we included other type of studies. However, low level evidences such as abstracts, conference articles, editorials, comments and expert opinions were excluded from the final list.

*Type of Participants*

We intended to include people with spinal cord injuries. 

*Type of Interventions*

Only studies that focus on various interventions use to control bowel and bladder functions in SCI were included and we excluded the studies on diet and medicines.

*Secondary Outcomes*

Any adverse effects reported in the included studies were considered as secondary outcomes.

*The studies Selection*

Two researchers independently screened the articles based on the inclusion criteria to determine their suitability. This was done mostly based on the abstracts and titles. If there was any sense of disagreement, a third researcher was enrolled. 

*Data Extraction and Management*

In this review, data extraction was based on population, intervention, comparison and outcomes (PICO). We tried to include duration of follow up, outcome assessed and also we reported any adverse effects of the mentioned treatment approaches. Some parameters such as number of selected subjects, lesion type, lesion level, procedure used, and final outcomes were summarized. 

*Quality Assessment and Determination of the Bias Risk*

The studies quality was evaluated by Pedro tool using. Actually Pedro scale was developed to measure methodological quality of randomized and quasi-randomized controlled trials in physical therapy. However, now it has been used to assess the quality of studies with different health care interventions such as exercise, psychological, behavioral interventions and medical and pharmacological interventions [23]. This scale has a high degree of reliability to assess the quality of various research studies. This scale comprises 11 items includes inclusion criteria and source, random allocation, allocation concealment, baseline comparability, subjects blinding, therapists blinding, assessment blinding, follow up, intention to treat analysis, between group comparison and point estimate and variability [23,24].

## Results

Based on the aforementioned key words, 100 paper were selected. Forty-three papers were selected after screening the papers based on titles and abstracts. Finally, 21 papers were selected in which 3 research were on abdominal muscles stimulation [21, 22, 25], one on tibial nerve stimulation [26], 8 papers on stimulation of sacral nerve root and conus medularis [16, 18, 27-32], 2 on stimulation and exercise combination [33, 34], 4 on brindley bladder control use [35-38] and 3 on sacralizotomy in combination with implantation of anterior sacral stimulator [39-41]. Tables 1-4 also show the quality of studies selected in this review article. As it can be seen, the quality of the papers on abdominal muscles stimulation various between 6 and 7. For the papers on stimulation of tibial nerve, the quality was 6. The quality of the papers on stimulation of sacral nerve root and conus medularis varied between 5 and 6. There were only two papers on stimulation and exercise combination with quality of 5-6. Most of the papers published on brindley bladder control had quality between 5 and 6.

**Table 1:** The brief review of the methods and results of the studies done on bowel and bladder management of SCI individuals

**References **	**Subjects **	**Procedure **	**Results**	**Quality assessment**
[25]	10 subjects with SCI participated in this study.	The subjects were divided into two groups 1) with stimulation 2) with placebo NMES of abdominal muscles. Stimulation done 25 min per day for 8 weeks.	NEMS significantly decreased forced vital capacity. This study showed that NMES of paralyzed abdominal muscles affect colonic transit.	7
[22]	Two able bodies man and 13 man with SCI (level ranging from C3 to L1) participated in this study.	A commercially available magnetic stimulator with MCS was used. Two protocols were employed 1) MS placed on trans abdominal and lumbosacral regions to check the effect of FMS on rectal pressure 2) 5 week stimulation to check the effect of FMS on total and segmental chronic transit time.	There was an increased in rectal pressure and a decreased in CTT by magnetic stimulation. FMS is able to stimulate colon and reduce CTT. It can be used to manage bowl in SCI.	6
[26]	Two subjects 1) 51 years old woman 2) 31 years old-both with paraplegic symptoms participated in this study.	TNS was used to stimulate tibial nerve. Unilateral stimulation was performed for four week (each time for 30 minutes). Then PTNS was repeated every two months for three times.	PTNS is an effective method for treatment of facial incontinence caused by partial spinal cord injury.	6
[21]	22 patients with chronic SCI were recruited in this study.	Three week functional magnetic stimulation was done. The colonic transit time assessment and Knowles-eccersly –scott symptom questionnaire were carried out for each patient before they received 3 week stimulation.	The improvement in bowel function showed that this method can be used to treat neurogical bowel dysfunction in spinal cord injury individuals.	7
[33]	20 male subjects with SCI at lumbar and lumbosacral regions participated in this study (age=15-30).	IFT with frequency of 4000 Hrz and frequency modulation between 50Hz and 100 was used to treat bladder incontinence. Specific exercises were given to strengthen the lower abdomen and bladder muscles.	IFT and exercise help to improve bladder function in the subjects. The results of this study support pervious finding that physiotherapeutic procedure have tremendous potential for achieving improvement in functional outcome in the subjects with inability to control bladder function.	5

Nero-electrical-muscle-stimulation (NEMS), Magnetic coils stimulation (MCS), Muscle stimulation (MS), Functional-magnetic-stimulation (FMS), Spinal cord injury (SCI), Colon transmit time (CTT), Tibial nerve stimulation (TNS), Posterior tibial nerve stimulation (PTNS), Interferential therapy (IFT)

**Table 2:** The brief review of the methods and results of studies done on bowel and bladder management of SCI individuals

**References **	**Subjects **	**Procedure **	**Results**	**Quality assessment**
[18]	Nine men and 2 women with SCI (6 were complete paraplegic one incomplete and other with lesion at C6).	They received radio linked implants to stimulate the S2, S3, S4 anterior roots. The follow up of the subjects varied between 2 months to 4 years.	The subjects could empty their bladder successfully and majority of them achieved continence.	6
[16]	500 SCI subjects were recruited in this study.	Subjects received anterior sacral root stimulation implanted for bladder control.	From 500 implanted, 479 survivors were using their implant. The time of follow up varied between 3 months and 16.1 years (mean 4 years) after implantation.	5
[28]	50 patients with SCI (38 men and 12 women) participated in this study.	Subjects received anterior sacral root stimulation and were followed for a period between 1 and 9 years.	49 are alive and 43 are regularly using their implants for micturition. 39 were very pleased, without significant reservation.	6
[29]	27 patients with complete suprasacral spinal cord injury participated in this study.	They were received introduced posterior sacral root rhizotomies from S2 to S5 in combination with implantation of an intradural finetech-Brindley bladder stimulator.	This method is a safe and effective produce in SCI patients.	6
[30]	12 subjects with complete suprasacral spinal cord injury with neurologic bladder and bowl participated in this study.	Annual cost of bladder and bowel with and without neuroprosthesis for a period of 10 years were evaluated.	Use of neuroprosthesis to control bowl and bladder has a significant influence to reduce bowl and bladder care cost.	5
[35]	68 male and 28 females with SCI lesions participated in this study.	Bridly-Fintech sacral anterior root stimulators combined with posterior sacral rhizotomies were implanted in these patients.	Of the 93 survival patients 83 used their implant. Bladder capacity increased from 206 ml preoperatively to 564 ml after operation. Erection was possible with electrical stimulation in 46 males. Sacral anterior root stimulation combined with sacral deaf fermentation is a good option to treat neurologic bladder in SCI patients.	6

Sacral (S), Spinal cord injury (SCI)

**Table 3:** The brief review of the methods and results of studies done on bowel and bladder management of SCI individuals

**References **	**Subjects **	**Procedure **	**Results**	**Quality assessment**
[36]	25 subjects with SCI, treated by Brindley technique, participated in this study.	Brindley technique was used based on section of posterior sacral nerve roots to control electro stimulation of anterior sacral nerve roots to empty bladder and facilitate erection and defection.	Acquisition of continence in 90% of subjects, bladder capacity increased successfully. Complete bladder emptying occurred in the majority of cases. Urinary tract infection decreased. Problems: Leaks, postoperative denervation sepsis, material or cable failure.	6
[39]	No information	Sacral rizotomy combined with implantation of anterior sacral root stimulator was done for the patients.	This method seems to be an effective method not only for the treatment of voiding dysfunction but also for defection and sexual disorder. The functional status of the patients increased significantly. 90% of patients gain satisfactory continence and their bladder capacity increased. This is a valuable method to restore bladder function in SCI suffering from hyperactive bladder.	5
[40]	10 SCI	Bladder function was compared pre and post operatively. Intradural sacral posterior rhizotomy combined with Intradural sacral anterior root stimulation was used for the patients.	Stimulation of S3 and S4 was mostly used to empty bladder (7 out of 10). Mean postoperative bladder capacity increased significantly. No major complication seen after operation. Autonomic hyperreflexia decreased but not suppressed by posterior sacral rhizotomy.	6
[34]	12 SCI with complete lesion (10 thoracic, 7 cervical). Time since implementation ranged from 3 months to 6 year.	Sacral anterior root stimulators were implanted after 2 years of injury.	Six patients achieved complete sacral evaluation with implant with no need for manual help. Total time for defecation reduced. This system helps SCI subjects to achieve complete unassisted defecation.	6
[37]	7 paraplegic patients participated in this study.	They obtained Brindley electro micturition sacral implant.	After stimulation, high activity was seen from transverse column to rectum. The greatest response was seen with stimulation of S3.	6
[31]	36 subjects, 22 female 14 male (age range 10-79) were selected for this study.	Sacral nerve stimulation was used in this study. The subjects were followed up for a period between 12 and 24 months. Number of incontinence episodes, maximum resting and squeeze anal canal pressure and quality of life were measured in this study.	29 subjects reported positive results: The number of incontinence episodes decreased from 7102 in 21 days. The maximum resting pressure and squeeze pressure improved after follow up period. Most of subjects reported an improvement in quality of life.	6

**Table 4:** The brief review of the methods and results of studies done on bowel and bladder management of SCI individuals

**References **	**Subjects **	**Procedure **	**Results**	**Quality assessment**
[32]	18 subjects with SCI (9 men, 9 women) participated in this study.	Sacral anterior nerve stimulation was used for these subjects. They were followed up for a period between 12 and 21 months post implantation. Outcome measure included: the numbers of bowl evaluation methods used, frequency of and time dedicated to bowel movements, constipation the wexner score.	The frequency of bowel movements significantly increased. In contrast time dedicated to bowel movements decreased (the difference was not significant). Constipation significantly decreased.	6
[41]	16 adults with SCI and history of bowel compliance were recruited in this study.	Sacral roots electrodes were implanted with rhizotomy at conus medularis. Finetech Brindley stimulator was used to stimulate the electrodes. The assessed parameters include occurrence of autonomic dysreflexia and quality of life.	Bowel program times reduced from 5.4 hour per weeks to 2 hour per week post operatively. The quality of life of the participants improved due to greater sense of independence, increased socialization. Greater control over their lives improved self-image and decreased feeling of depression.	6
[38]	93 patients with SCI and 70 SCI as control group.	The quality of life of the subjects received Brindley procedure evaluated in this study. The Qualiven questionnaire, SF-36 questionnaire and multiple choice questions about urinary continence and tract infection were sent to 93 patients with Brindley stimulator.	Urinary problems score was 78% and 40% for the patients with Brindley stimulator and control, respectively. Urinary tract infection decreased in the subjects used stimulator. It has been shown that Brindley stimulator for SCI improves quality of life, continence and urinary tract infection compared to match control group.	5
[27]	177 SCI patients participated in this study.	These patients underwent percutaneous nerve stimulation. The subjects were followed for a period of 6 years.	It has been shown that sacral nerve stimulation is a simple safe and minimally invasive technique with excellent results. Michigan wexner incontinence score decreased significantly. The infection rate was 1.6%.	6

Tables 1-4 summarized the methods and the outcomes of the studies. As it can be seen from these tables, the number of subjects was high in some studies. Moreover, most of the subjects were monitored for a long period of time. The results of the reviewed studies can be summarized as follow:

Neuro-electrical-muscle-stimulation (NEMS) significantly decreased force dutal capacity and influence colonic transit,

Functional-magnetic-stimulation (FMS) is able to stimulate colon and reduce colon transmit time (CTT),

Functional magnetic stimulation can be used to treat neurological bowel dysfunction in SCI,

Transcutaneous electrical nerve stimulation (TENS) and exercise helps to improve function of bladder,

Posterior sacral root rhizotomy seems to be a safe and effective method,

Sacral rhizotomy combined with implantation of anterior sacral root stimulation is valuable method to restore bladder function in SCI suffer from hyperactive bladder, Sacral anterior root stimulator helps SCI to achieve complete transited defection, and Most of the subjects received sacral nerve stimulation reported an improvement in quality of life. 

## Discussion

There is no doubt that most of the subjects with spinal cord injury missed their abilities to control their bowel and bladder functions. This limits their daily performance and also their social activities. Various methods have been used to restore the abilities of these subjects to control their bowel and bladder function. Functional electrical stimulation (FES) is a method recommended in this regard. Various methods of stimulation have being used for SCI subjects. The purpose of this review was to evaluate the published literature in this regard. 

*Stimulation of Tibial Nerve*

There was only one study on tibial nerve stimulation to control bowel and bladder function [26]. The quality of this study based on Pedro scale was 6. The results of this study showed that it was an effective method to treat bowel and bladder function insufficiency in the subjects with partial spinal cord injury [26]. It should be emphasized that stimulation was performed for four weeks and were repeated every two months for three times, however, as the number of studies done on this topic was small, it is not easy to recommend this method (approach) to manage bowel and bladder function in this group of subjects.

*Stimulation of Sacral Nerve Root and Conus Medularis*

This is another approach to manage bowel and bladder functions in SCI. There were 7 studies published on this topic. Valleys *et al*., [32] showed that using this method frequency of bowel movement significantly increased and time dedicated to bowel movement decreased. In the study of Michelson *et al*., [27] on 177 patients, the subjects were monitored for a period of 6 years. They concluded that sacral nerve stimulation is a simple method with minimal side effects, which provides excellent output to control bowel and bladder functions. Due to the number of subjects participated in these studies, and duration of follow up, it can be concluded that stimulation of sacral nerve root is a good approach to manage bowel and bladder function in SCI subjects.

*Stimulation of Abdominal Muscles*

As it can be seen in Table 1, there were 3 studies on abdominal muscles stimulation of SCI bowel function in individuals. The number of the subjects in these studies varied between 2 and 22 patients. The results of these studies showed that the subjects had an improvement in bowel function. Moreover, it influenced the colonic transit time. 

*Brindley Bladder Control*

This is the other method recommended for SCI subjects. There were 4 studies published on this topic. Based on the results of the study of Egon *et al*., [35] bladder capacity increased after use of this method. Errection was also possible follow the use of this approach. Increased bladder capacity, complete bladder empting in most of the subjects, decreased in urinary tract infection, increased in activity of transverse column to rectum and improved quality of life were the advantages mentioned for use of this method [35, 36,37, 38].

*Sacralizotomy with Anterior Sacral Stimulation*

The results of the most studies showed that this method is an effective approach to help SCI subjects to manage their bowel and bladder function. However, the main complication which has been reported by using of this method, may be its influence on sexual function and erection in male SCI individuals. Schurch *et al*., [40] showed that bladder capacity increased significantly with no major complications. The other advantages mentioned for this approach includes decreased in bowel program time, improved quality of life, improved socialization, decreased feeling of depression and improved self-image [39, 41].

*Stimulation and Exercise*

The other approach which consists of a stimulation and exercise combination was also recommended by Buhroo *et al*., [33]. The quality of their study was acceptable as 20 male subjects with SCI participated in this study. Some exercise was used to strength the lower abdomen and bladder muscles. Although there was only one study on this topic which confirmed that the physiotherapeutic procedure has tremendous potential to achieve improvement in control of bowel and bladder function.

From above mentioned studies and based on the results of quality assessment, it can be concluded that bowel and bladder control is not a big issue for this group of subjects in a social acceptable manner. Although stimulations of the muscles and nerve seems to be an improved approach to control bowel and bladder function, there are some issues that need to be resolved. Decreasing the stimulation electrodes infection risk, decreasing the side effects of this procedure on sexual performance of SCI subjects, and decreasing the threshold of stimulation are some problems which should be solved in future studies. 

## Conclusion

Based on the results of the studies done on functional electrical stimulation use for bowel and bladder functions management, this method seems to be successful for SCI subjects. Improved quality of life, socialization, and increased bowel and bladder functions are some advantages of using this procedure. It seems that strengthening of abdominal muscles, stimulation of these muscles, and combination of abdominal muscles stimulation and strengthening of abdominal muscles can be used as the first step to manage bowel and bladder functions in SCI subjects. Although other methods use have been also recommended for these subjects, it seems to be costlier and required especial facilities. Last but not least is that most of the studies done on limited number of subjects and mostly on male participants. Also no comparison was done between the outputs of various treatment approaches. Therefore, it is recommended to do a big study to cover the mentioned important comparisons.

## Declarations

### Ethics approval and consent to participate:

This study was supported by Shiraz University of Medical Sciences (SUMS) (IR.SUMS.REC.1397.587)

### Conflict of Interests:

None declared.

### Consent for publication:

Both authors agree with the publication of this article

### Funding:

This study was supported by Shiraz University of Medical Sciences (SUMS)

### Authors'' contributions:

MTK: Concept and design; AFA: Analysis and interpretation; MTK: Data Collection; MTK & AFA: Writing the Article; MTK: Final Approval.

### Acknowledgements:

Not applicable

## References

[1] Wyndaele M, Wyndaele JJ (2006). Incidence, prevalence and epidemiology of spinal cord injury: what learns a worldwide literature survey?. Spinal Cord.

[2] Surkin J, Gilbert BJ, Harkey HL 3rd, Sniezek J, Currier M (2000). Spinal cord injury in Mississippi Findings and evaluation, 1992-1994. Spine (Phila Pa 1976).

[3] O’Connor PJ (2005). Prevalence of spinal cord injury in Australia. Spinal Cord.

[4] Maharaj JC (1996). Epidemiology of spinal cord paralysis in Fiji: 1985-1994. Spinal Cord.

[5] Colombo G, Wirz M, Dietz V (2001). Driven gait orthosis for improvement of locomotor training in paraplegic patients. Spinal Cord.

[6] Karimi M, Omar AH, Fatoye F (2014). Spinal cord injury rehabilitation: which way forward?. NeuroRehabilitation.

[7] Karimi MT (2013). Robotic rehabilitation of spinal cord injury individual. Ortop Traumatol Rehabil.

[8] Karimi MT, Amiri P, Esrafilian A, Sedigh J, Fatoye F (2013). Performance of spinal cord injury individuals while standing with the Mohammad Taghi Karimi reciprocal gait orthosis (MTK-RGO). Australas Phys Eng Sci Med.

[9] Stolov W, Clowers M (1981). Handbook of Severe Disability: A Text for Rehabilitation Counselors. Other Vocational Practitioners, and Allied Health Professionals.

[10] Creasey GH, Grill JH, Korsten M, U HS, Betz R, Anderson R (2001). An implantable neuroprosthesis for restoring bladder and bowel control to patients with spinal cord injuries: a multicenter trial. Arch Phys Med Rehabil.

[11] Stiens SA, Bergman SB, Goetz LL (1997). Neurogenic bowel dysfunction after spinal cord injury: clinical evaluation and rehabilitative management. Arch Phys Med Rehabil.

[12] Lynch AC, Antony A, Dobbs BR, Frizelle FA (2001). Bowel dysfunction following spinal cord injury. Spinal Cord.

[13] Lynch AC, Wong C, Anthony A, Dobbs BR, Frizelle FA (2000). Bowel dysfunction following spinal cord injury: a description of bowel function in a spinal cord-injured population and comparison with age and gender matched controls. Spinal Cord.

[14] Ebert E (2012). Gastrointestinal involvement in spinal cord injury: a clinical perspective. J Gastrointestin Liver Dis.

[15] Anderson KD (2004). Targeting recovery: priorities of the spinal cord-injured population. J Neurotrauma.

[16] Brindley GS (1994). The first 500 patients with sacral anterior root stimulator implants: general description. Paraplegia.

[17] Brindley GS, Rushton DN (1990). Long-term follow-up of patients with sacral anterior root stimulator implants. Paraplegia.

[18] Brindley GS, Polkey CE, Rushton DN (1982). Sacral anterior root stimulators for bladder control in paraplegia. Paraplegia.

[19] Brindley GS, Polkey CE, Rushton DN, Cardozo L (1986). Sacral anterior root stimulators for bladder control in paraplegia: the first 50 cases. J Neurol Neurosurg Psychiatry.

[20] Krassioukov A, Eng JJ, Claxton G, Sakakibara BM, Shum S (2010). Neurogenic bowel management after spinal cord injury: a systematic review of the evidence. Spinal Cord.

[21] Tsai PY, Wang CP, Chiu FY, Tsai YA, Chang YC, Chuang TY (2009). Efficacy of functional magnetic stimulation in neurogenic bowel dysfunction after spinal cord injury. J Rehabil Med.

[22] Lin VW, Nino-Murcia M, Frost F, Wolfe V, Hsiao I, Perkash I (2001). Functional magnetic stimulation of the colon in persons with spinal cord injury. Arch Phys Med Rehabil.

[23] Maher CG, Sherrington C, Herbert RD, Moseley AM, Elkins M (2003). Reliability of the PEDro scale for rating quality of randomized controlled trials. Phys Ther.

[24] de Morton NA (2009). The PEDro scale is a valid measure of the methodological quality of clinical trials: a demographic study. Aust J Physiother.

[25] Hascakova-Bartova R, Dinant JF, Parent A, Ventura M (2008). Neuromuscular electrical stimulation of completely paralyzed abdominal muscles in spinal cord-injured patients: a pilot study. Spinal Cord.

[26] Mentes BB, Yüksel O, Aydin A, Tezcaner T, Leventoğlu A, Aytaç B (2007). Posterior tibial nerve stimulation for faecal incontinence after partial spinal injury: preliminary report. Tech Coloproctol.

[27] Michelsen HB, Thompson-Fawcett M, Lundby L, Krogh K, Laurberg S, Buntzen S (2010). Six years of experience with sacral nerve stimulation for fecal incontinence. Dis Colon Rectum.

[28] Arnold EP, Gowland SP, MacFarlane MR, Bean AR, Utley WL (1986). Sacral anterior root stimulation of the bladder in paraplegics. Aust N Z J Surg.

[29] Koldewijn EL, Van Kerrebroeck PE, Rosier PF, Wijkstra H, Debruyne FM (1994). Bladder compliance after posterior sacral root rhizotomies and anterior sacral root stimulation. J Urol.

[30] Creasey GH, Dahlberg JE (2001). Economic consequences of an implanted neuroprosthesis for bladder and bowel management. Arch Phys Med Rehabil.

[31] Holzer B, Rosen HR, Novi G, Ausch C, Hölbling N, Schiessel R (2007). Sacral nerve stimulation for neurogenic faecal incontinence. Br J Surg.

[32] Vallès M, Rodríguez A, Borau A, Mearin F (2009). Effect of sacral anterior root stimulator on bowel dysfunction in patients with spinal cord injury. Dis Colon Rectum.

[33] Buhroo M, Hussain S (2011). Role of IFT in Rehabilitation of Neurogenic Bladder in Patients with Traumatic Spinal Cord Injuries. JK- Practitioner.

[34] MacDonagh RP, Sun WM, Smallwood R, Forster D, Read NW (1990). Control of defecation in patients with spinal injuries by stimulation of sacral anterior nerve roots. BMJ.

[35] Egon G, Barat M, Colombel P, Visentin C, Isambert JL, Guerin J (1998). Implantation of anterior sacral root stimulators combined with posterior sacral rhizotomy in spinal injury patients. World J Urol.

[36] Colombel P, Egon G, Isambert JL (1992). Electrostimulation des racines sacrées antérieures chez le blessé médullaire (bilan des 25 premiers cas) [Electrostimulation of anterior sacral nerve roots in spinal cord injury patients (evaluation of the 1st 25 cases)]. Prog Urol.

[37] Binnie N, Smith A, Creasey G, Edmond P (1990). Motility effects of electrical anterior sacral nerve root stimulation of the parasympathetic supply of the left colon and anorectum in paraplegic subjects. Neurogastroenterology & Motility.

[38] Martens FM, den Hollander PP, Snoek GJ, Koldewijn EL, van Kerrebroeck PE, Heesakkers JP (2011). Quality of life in complete spinal cord injury patients with a Brindley bladder stimulator compared to a matched control group. Neurourol Urodyn.

[39] Vignes JR, Bauchet L, Ohanna F (2007). Dorsal rhizotomy combined with anterior sacral root stimulation for neurogenic bladder. Acta Neurochir Suppl.

[40] Schurch B, Rodic B, Jeanmonod D (1997). Posterior sacral rhizotomy and intradural anterior sacral root stimulation for treatment of the spastic bladder in spinal cord injured patients. J Urol.

[41] Kachourbos MJ, Creasey GH (2000). Health promotion in motion: improving quality of life for persons with neurogenic bladder and bowel using assistive technology. SCI Nurs.

